# Fungal Strategies to Evade the Host Immune Recognition

**DOI:** 10.3390/jof3040051

**Published:** 2017-09-23

**Authors:** Marco J. Hernández-Chávez, Luis A. Pérez-García, Gustavo A. Niño-Vega, Héctor M. Mora-Montes

**Affiliations:** 1 Departamento de Biología, División de Ciencias Naturales y Exactas, Campus Guanajuato, Universidad de Guanajuato, Noria Alta s/n, col. Noria Alta, C.P., Guanajuato Gto. 36050, México; Mgunsandroses1000@hotmail.com (M.J.H.-C.); gustavo.nino@ugto.mx (G.A.N.-V.); 2 Unidad Académica Multidisciplinaria Zona Huasteca, Universidad Autónoma de San Luis Potosí, Romualdo del Campo 501, Fracc. Rafael Curiel, C.P., Cd. Valle SLP. 79060, México; luisantonio.perez@uaslp.mx

**Keywords:** fungal pathogen, cell wall, immune system, dimorphism, *Aspergillus*, *Cryptococcus*, *Candida*, *Sporothrix*, *Paracoccidioides*, phagocytosis

## Abstract

The recognition of fungal cells by the host immune system is key during the establishment of a protective anti-fungal response. Even though the immune system has evolved a vast number of processes to control these organisms, they have developed strategies to fight back, avoiding the proper recognition by immune components and thus interfering with the host protective mechanisms. Therefore, the strategies to evade the immune system are as important as the virulence factors and attributes that damage the host tissues and cells. Here, we performed a thorough revision of the main fungal tactics to escape from the host immunosurveillance processes. These include the composition and organization of the cell wall, the fungal capsule, the formation of titan cells, biofilms, and asteroid bodies; the ability to undergo dimorphism; and the escape from nutritional immunity, extracellular traps, phagocytosis, and the action of humoral immune effectors.

## 1. Introduction

Fungi can be a common and constant threat to human, animal, and plant health, causing a wide range of mycoses. Although superficial mycoses are regarded as low-concern infections in humans, subcutaneous and systemic mycoses are often related to high morbidity and mortality rates, especially in patients with temporal or permanent impairments in the immune response [1]. There is no doubt that the human immune system has developed key strategies to effectively control these organisms, usually ensuring the outcome will be in our favor. After entering the host, the first line of defense against the invading fungal cells are phagocytic cells of the innate immune system. Phagocytes are very efficient at recognizing fungi, mainly via interaction with the wall, which is composed of molecules that are not synthesized by the host and, therefore, are sensed as pathogen-associated molecular patterns (PAMPs) by different pattern recognition receptors (PRRs) [2]. Since polysaccharides are the most abundant components of the cell wall, we can expect that they will be the main PAMPs of the fungal cell. Indeed, almost all the fungal cell wall polysaccharides can be recognized by PRRs, and stimulate immune responses [3]. These can lead to a crosstalk with components of the adaptive branch of the immune system, which helps to establish a protective anti-fungal immune response. However, fungi have the ability to fight back, developing strategies to escape from the immune response. Here, we summarize the main and most studied mechanisms that fungal cells use to evade the host immunosurveillance processes.

## 2. The Fungal Cell Wall: Composition and Organization

The fungal cell wall is a dynamic organelle essential for cell viability, morphogenesis, and pathogenesis. It has the ability to change its composition and architecture due to the influence of environmental conditions and stress; therefore, this structure provides these organisms with the opportunity to adapt for survival in harsh conditions [2]. For many fungal cells, such environmental variations produce extreme changes in their ecology, driving them from a saprophytic to a parasitic lifestyle. As part of the adaptation process, the cell wall conserves some basic components found in almost all members of the fungi kingdom, while other components have suffered divergent evolution and are regarded as species-specific molecules [4]. Due to the natural distribution in the fungal cell, walls are the natural boundary between fungi and the extracellular space. The cell wall composition and structure are influenced by changes in the external environment sensed by the cell [2,3,5]. The composition and structure of the wall will eventually determine whether the fungus survives or not. Indeed, in pathogenic fungi, the wall plays a key role during infection [2,5,6,7]. The recognition of cell surface components, the wall in most of the fungal pathogens, is essential to trigger the host’s immune response; and the outcome of such recognition (fungal death or establishment of an infection) will depend on the PRR–PAMP interactions.

### 2.1. The Support: Polysaccharides

Polysaccharides comprise over 90% of the wall components and are the molecular scaffold that supports proteins, lipids, and superficial components. They make a strong, yet plastic, interphase that determines how the fungus ultimately interacts with its surroundings [2,5,6,7]. In most fungi, the cell wall polysaccharides are found as layers, although all components of the different layers are linked. Based on their solubility properties during extraction, cell wall polysaccharides are present as alkali-insoluble or alkali-soluble carbohydrates. The alkali-insoluble polysaccharides are arranged into a fibrillar skeleton, which defines the innermost layer of the wall, and is mainly composed of branched β-1,3-glucan cross-linked to microfibers of chitin by β-1,4-linkages, and assembled into microfibrils. The β-1,3-glucan-to-chitin proportion changes according to the fungal species, and in some cases according to the morphotype of the fungal cell [5,8]. The alkali-soluble matrix is composed of amorphous polysaccharides, whose chemical composition also changes according to the fungal species, and are found in the outer layer of the wall [5]; therefore, this is a more heterogeneous layer. The best studied outer layer in fungal pathogens is that of *Candida albicans*, which comprises highly mannosylated glycoproteins, with α- and β-linked oligomannosyl residues [5,7,8]. Unlike *Candida*, *Aspergillus fumigatus* has mannan chains that are directly bound to the cell wall glucan–chitin core, with no intermediary peptides in the cell wall of hyphae [6]. However, the major decorative polysaccharides present in the *A. fumigatus* outermost layer are galactomannan, galactosaminogalactan, and α-1,3-glucan [6]. The latter is an amorphous polysaccharide that plays an important role on the cell wall of several pathogenic fungi. Besides *A. fumigatus*, it is also found in the *Cryptococcus neoformans* yeast cell wall, anchoring the large capsule, composed of glucuronoxylomannan (GXM) and galactoxylomannan (GalXM), to the main inner fibrillar wall. The α-1,3-glucan is also present as the outermost layer in the yeast phase of the dimorphic fungi *Histoplasma capsulatum*, *Paracoccidioides* spp., and *Blastomyces dermatitidis* [5,9].

The general arrangement of the fungal cell wall places the fibrillar structural elements closer to the membrane, providing mechanical support to the whole structure, while the most hydrophobic and amorphous polymers are arranged on the outside [3], an organization that has important implications for the interaction of the fungal cells with the host, including components of the immune system. The cell wall polysaccharides are the main fungal PAMPs, and as expected, they are sensed by PRRs and trigger an immune response [3]. However, fungal cells have evolved strategies to avoid recognition by immune cells, modulating the amount of certain cell wall components or the accessibility of them on the fungal cell surface.

**Figure 1:**
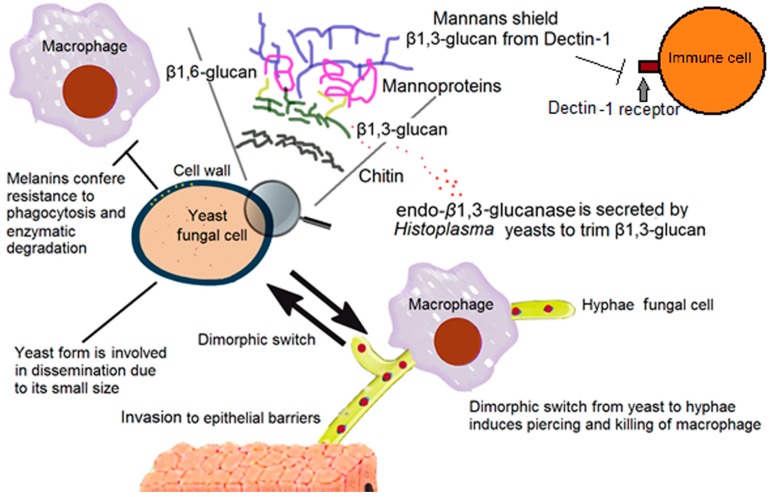
Components of the cell wall that help fungi to evade the immune system. The outer layer of mannans of many fungal cells shields the highly immunogenic β-1,3-glucan from Dectin-1 receptor, precluding the activation of effector mechanisms in immune cells. In the case of *Histoplasma* cells, endo- β-1,3-glucanase is released to trim β-1,3-glucans from the cell and prevents its recognition via Dectin-1 receptor. Melanin is distributed into the cell wall of some fungal pathogens and contributes to resistance to phagocytosis and enzymatic degradation by macrophages. Finally, the reversible, dimorphic switch between yeast and hyphae confers special traits to each form that helps the pathogen to disseminate and invade the host.

As mentioned before, β-1,3-glucan and chitin are the polysaccharides responsible for the structural maintenance of the fungal cell wall; and therefore, are of extreme importance for the fungal survival. The β-1,3-glucan is a highly immunogenic molecule, and unlike other fungal cell wall polysaccharides, it has its own very specific PRR on the surface of the host’s immune cells, named Dectin-1 [10]. Both in vitro and in vivo studies have shown that Dectin-1 mediates a variety of antifungal cellular responses, such as phagocytosis, respiratory burst, and production of cytokines and chemokines, contributing to a strong inflammatory immune response. This immune response is, in fact, the product of a collaborative work with Toll-like receptor 2 (TLR2) [11], which, together with other Toll-like receptors, contributes to the recognition of different cell wall-decorating polysaccharides, such as GXM, α-1,4-glucans, and phospholipomannans, depending on the invading fungal species [3]. Dectin-1 binds to several fungal species such as *C. albicans*, *Coccidioides immitis*, and *A. fumigatus* [12], and potentially to any β-1,3-glucan-containing organism. To bypass the encounter with immune cells, fungi have developed a “hiding” strategy by covering β-1,3-glucan and chitin with different molecules (Figure 1). Although different covers have evolved in different fungal pathogens, they all converge to the same result: shielding of PAMPs. Dectin-1 has shown poor reactivity against yeast cells of *C. albicans, C. guilliermondii, C. parapsilosis*, and *C. glabrata*, and this has been related to β-1,3-glucan shielding by an outer mannan layer. Indeed, perturbation of the cell wall integrity and β-1,3-glucan exposure led to Dectin-1 binding, triggering macrophage activation and cytokine production by innate immune cells [13,14,15,16,17,18,19,20]. Therefore, the mannan layer in these fungal species works as a protective shield that prevents interaction of β-1,3-glucan with immune receptors. In the same line, the presence of α-1,3-glucan in the outermost layer of the cell wall of *H. capsulatum* yeast covers β-1,3-glucan, precluding its recognition from Dectin-1 [21]. Depletion of the α-1,3-glucan synthesis, by reducing the abundance of the *H. capsulatum* α-1,4-amylase (AMY1) transcript, reduced the cell wall α-1,3-glucan content and fungal virulence [22]. Additionally, a recent study showed that *Histoplasma* yeast cells secrete an endo-β-1,3-glucanase, Eng1, which plays a role on trimming β-glucan segments exposed on the fungal cell surface, minimizing potential recognition via Dectin-1, with the consequent reduction in the level of proinflammatory cytokines, thus improving the ability of *Histoplasma* to escape from detection by host macrophages (Figure 1) [23]. Table 1 summarizes some of the molecules involved in evasion of the host immune response.

On the other hand, chitin interacts with different PRRs, and this interaction occurs in a size-dependent manner. Both big (70–100 µm in diameter) and small (<2 µm) chitin particles are inert, and do not trigger any immune reaction; while intermediate-sized chitin particles (40–70 µm in diameter) induce a proinflammatory response, triggering the production of TNFα and IL-17, and small fragments (2 to 10 µm) induce an anti-inflammatory response through the mannose receptor, co-localizing with the nucleotide-binding oligomerization domain-containing protein 2 (NOD2) and TLR9, leading to IL-10 secretion [36,37]. These findings lead us to speculate that, during the course of infection, and on the assumption that intermediate-sized particles of chitin have been produced during the first stages of the infection, inflammation and destruction of the fungus are induced to promote the elimination of the invading organism, but once the fungus has been killed, the chitin particles found will mostly be small, which will then trigger an anti-inflammatory response through the production of IL-10, mitigating inflammation and therefore avoiding excessive tissue injury [36]. The increment in the chitin content, as a consequence of exposure to sublethal concentrations of caspofungin, has a negative impact on the ability of fungal cells to induce cytokine production [38], indicating that upregulation of these wall components might provide an advantage to fungi seeking to escape from interaction with immune cells.

### 2.2. Other Cell Wall Components

Melanins are high molecular weight hydrophobic biological pigments, made by the oxidative polymerization of phenolic and/or indolic compounds [24]. They are present as negatively charged amorphous molecules; their insoluble nature makes them difficult to study, so their molecular structure remains unknown [25]. In fungi, they are present within the cell wall, contributing to its mechanical strength, reduced susceptibility to enzymatic degradation, and resistance to UV light (Figure 1 and Table 1) [24]. Melanized fungi have an augmented resistance to phagocytosis compared to non-melanized cells, as reported for *C. neoformans* and *Paracoccidioides brasiliensis* [9,25]. In *C. neoformans*, such resistance to phagocytosis has been related to changes in the fungal cell surface charge that may contribute to inhibiting phagocytosis. It has also been related to significantly reduced nitric oxide production by macrophages in *C. neoformans*, *Aspergillus* spp., *Sporothrix schenckii*, and *Fonsecaea pedrosoi* [25].

In *C. albicans* and *C. neoformans*, melanin is formed by the cross-linking of phenolic compounds obtained from host tissues. These organisms use a cell wall laccase/tyrosinase to generate l-(3,4)-dihydroxyphenylalanine (DOPA) from tyrosine, then DOPA is subsequently oxidized to dopaquinone [39]. After an intramolecular nucleophilic attack cyclo-DOPA is produced and once it is oxidized gives rise to dopachrome. The dopachrome is tautomerized to form dihydroxyindoles, which polymerize to form eumelanin. This pathway may also incorporate sulfur to produce pheomelanins, which are not brown, but red or yellow in color [40]. During melanin synthesis, the covalent bonds binding the individual phenolic compounds are randomly formed by a free radical reaction and then, once inside the polymerized molecule, form numerous different bonds. This diversity in their bonds and the random manner in which they occur make melanin structurally complex and difficult to digest by enzymes [41,42]. Most other fungi synthesize melanin via 1,8-dihydroxynaphthalene (DHN) pathway, instead of the l-DOPA pathway. In the DHN pathway, the precursor molecule is the endogenous acetyl-CoA or malonyl-CoA, which is used to form 1,3,6,8-tetrahydroxynaphthalene, catalyzed by a polyketide synthase. Then, after a series of reduction and dehydration reactions, DHN is produced. Finally, polymerization of DHN produces melanin [43,44]. Interestingly, although some fungi as *C. neoformans* rely only on the l-DOPA pathway to synthesize melanin, many other fungi, such as *S. schenckii*, produce melanin using both pathways [45]. The melanization of the cell wall in *C. albicans* occurs during the infection process, although its importance as a possible virulence factor has not been fully established [46]. So far, it is known that melanin is externalized as melanosomes, which may be found free or slightly bound to the outside of the plasma membrane; and this process depends on the structures involved in the synthesis and remodeling of chitin [47]. Despite this, l-DOPA and DHN pathways are alike in that indolic and/or phenolic precursors are polymerized into melanin; they differ in how the process is achieved, because the l-DOPA pathway is considered to occur spontaneously after the initial oxidation by laccase—unlike the DHN pathway, in which every step is enzymatically catalyzed. Furthermore, the melanin synthesized via the l-DOPA pathway contains carbon, oxygen, and nitrogen, while that produced via DHN pathway does not contain nitrogen [42].

Melanin seems to be a central element of *Aspergillus* against the host defense, as studies indicate that DHN-melanin, covering the conidia, inhibits the phagolysosome acidification of alveolar macrophages. This effect is much stronger in *A. fumigatus* and *A. flavus* than in *A. nidulans* and *A. terreus* [48].

Cell wall proteins play important roles in the fungal–host interaction, intervening in adhesion to different surfaces, as protection against harmful environmental hazards, disguising from phagocytes, and even in the cell wall remodeling during growth, morphological changes, and modifying cell wall properties during adaptation to changes in the external environment. Hydrophobins are wall proteins that can self-assemble onto the surface of filamentous fungi, producing a hydrophobic edge between the fungal cell and their environment. They form a monolayer (the rodlet) around hyphae, fruiting bodies, and conidia [5]. In medically relevant fungi, the only well-characterized hydrophobins are those found on the *A. fumigatus* conidial surface, known as RodA and RodB [49]. RodA is an essential component for rodlet formation, unlike RodB (Table 1). However, RodB seems to play a role in the structure of the conidial cell wall, since the surface of the conidia of the Δ*rodA* mutant is granular due to the lack of the rodlet layer, but the double mutant Δ*rodA* Δ*rodB* showed conidia with an amorphous layer on the surface. Also, *Aspergillus* rodletless mutants are more sensitive to killing by alveolar macrophages, suggesting that killing molecules produced by the phagocytic cell following activation, are impeded of crossing the hydrophobic rodlet layer [49,50].

## 3. Fungal Capsule

Besides the cell wall, some microbes have surface structures that act as a physical barrier to protect them from the hostile medium. These structures can mediate binding to tissues, lead to dissemination, and determine whether the microorganism is detected by the host or evades the surveillance mechanisms. Polysaccharide capsules (PC) are present in both Gram-positive and Gram-negative bacteria and are a well-studied virulence factor that provide defense against the host immunity [51]. PC has been described in a limited number of fungi. Although *H. capsulatum* was given its name because it was thought it had a capsule, it is now well known that it lacks one [52]. On the other hand, the opportunistic pathogen *C. neoformans*, a fungus responsible for half a million cases of meningitis each year in HIV/AIDS patients globally, has a peerless and large capsule, which is considered to be one of its main virulence factors [53]. This PC is composed mainly of GXM, a small portion of GalXM, and 99% of its total weight is water [54]. Capsule carbohydrates can be attached to the cell, forming a physical surface, or can be released to the medium as an exo-polysaccharide [55]. In both cases, it seems to be beneficial for the fungal cell (Table 2).

**Figure 2:**
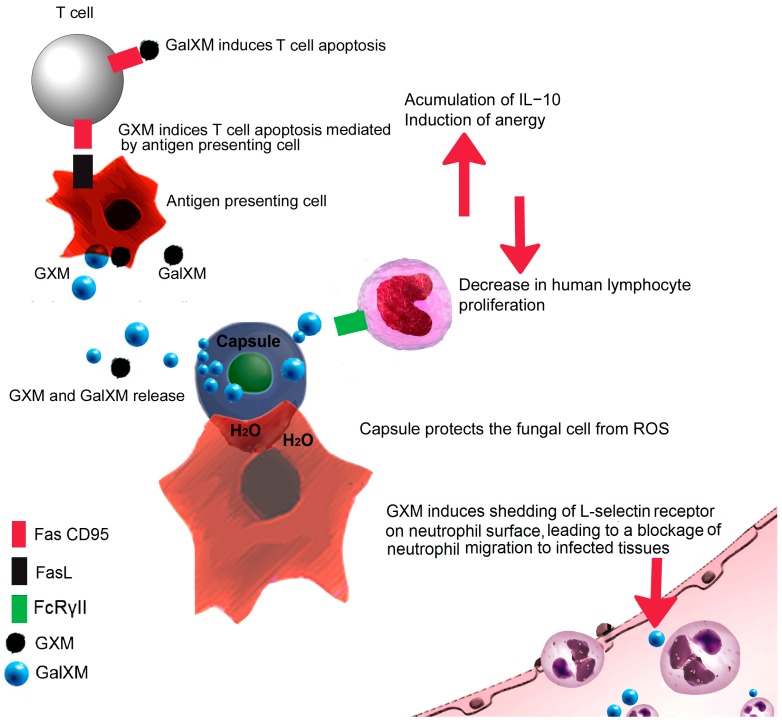
Mechanisms whereby the *Cryptococcus neoformans* capsule subverts the immune system. Capsule components are released into the extracellular space and directly induce T-cell apoptosis or cell death mediated by antigen presenting cells. Capsule components also decrease human lymphocyte proliferation and induce shedding of l-selectin and TNFα receptor on the surface of neutrophils, blocking their attachment to the endothelial surface and, therefore, migration to infected tissue is reduced. Once *C. neoformans* is phagocytosed, capsule enlargement occurs and protects the fungal cell from ROS-mediated killing.

The *C. neoformans* capsule has strong anti-phagocytic properties, suppresses T lymphocyte proliferation, induces the anti-inflammatory cytokine IL-10, and affects the production of important proinflammatory cytokines, such as TNFα and IL-1β (Figure 2) [56]. Even though these properties seem to act in favor of the fungal pathogen, an important amount of yeasts are phagocytosed during the course of cryptococcosis, [67], meaning that there are mechanisms that mask or undermine the anti-phagocytic nature of the capsule. One of these mechanisms is the phagocytosis induced by anti-GXM mAbs, which can bind the capsule and work like phagocytic opsonins [53].

*C. neoformans* GXM, a mannose-rich molecule, is recognized by the mannose receptor and induces uptake by dendritic cells. However, the *C. neoformans* capsule produces immunomodulation by the secretion of soluble mannoproteins that interact with mannose receptor [68]. Furthermore, GXM induces shedding of L-selectin and TNFα receptors from the surface of neutrophils, preventing the attachment to endothelial surfaces, resulting in a migration blockage of neutrophils from vessels to infected tissues, a phenomenon that explains the reduced cell infiltration in tissues of patients suffering from disseminated cryptococcal infections (Figure 2) [69].

Phagocytosis is not enough to kill capsular *C. neoformans* strains; they can survive within the phagocytic cell and sustain replication using the phagocytic environment [55].The observation that non-capsulated *C. neoformans* mutants cannot replicate inside macrophages strongly suggests that the capsule is critical for cell survival [67]. It was also demonstrated that macrophages preferentially phagocytize cells with small capsules [70]. Inside the phagocyte, the capsule protects the fungal cell from ROS, radicals and anti-fungal molecules, such as defensins, which are produced by the macrophage during phagocytosis [71].

The size of the *C. neoformans* capsule can vary, depending on the environmental stimuli, such as osmotic pressure and sugar content. The capsule enlargement is associated to resistance to oxygen species, antimicrobial peptides and the antifungal drug amphotericin B, demonstrating that the capsule enlargement helps the pathogen to survive inside the phagosome [72]. In addition, *C. neoformans* capsule increases size after 24 h of infection, limiting further phagocytosis in vivo, suggesting that early interaction between *C. neoformans* and macrophages is critical for the outcome of the infection [70]. This capsule enlargement can also affect the effector properties of complement activation, preventing the interaction of C3 with phagocytic receptors [73].

Both GXM and GalXM can induce apoptosis in different cells [74]. Accumulation of GXM in macrophages leads to induction of FasL, promoting apoptosis in T cells that express Fas [75]. Moreover, GalXM binds directly to galectins and induces apoptosis of T cells, via caspase 8 activation and the consequent DNA fragmentation (Figure 2) [75,76].

Finally, there are reports indicating that capsular GXM engages with FcRγII of monocytes, dendritic cells, and macrophages, inducing immunosuppression, and decreasing the proliferation of human lymphocytes [77,78]. This negative effect on the immune response is also related to the accumulation of IL-10, as previously mentioned; downregulation of Th1 cytokines; and enhancement of IL-4 production [79,80]. Encapsulated *C. neoformans* strains also have a negative effect on maturation markers of antigen-presenting dendritic cells, leading to an impaired T-cell-mediated response [57].

## 4. Titan Cells

**Figure 3:**
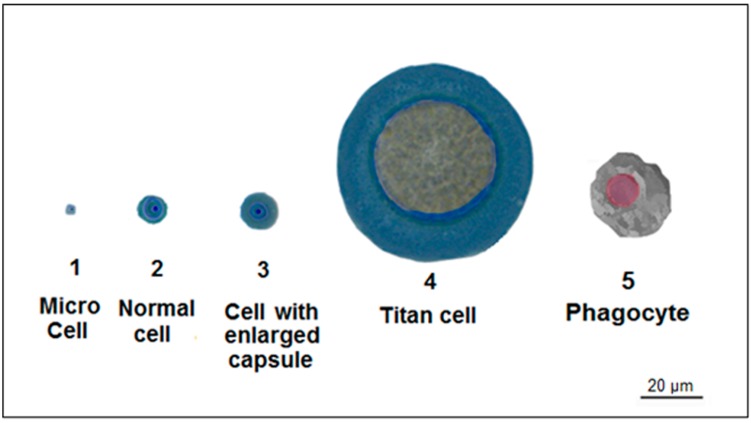
Different cell populations found in lungs of infected mice with *C. neofromans*. Yeasts with different capsule sizes, microforms, regular size cells, and titan cells, which are larger than phagocytes, are present during lung infection in mice.

*C. neoformans* does not form hyphae during infection but possesses other unique mechanisms that influence its virulence. As previously described, it has a capsule and its shape and size can be modulated for its benefit. Furthermore, *C. neoformans* can also modulate the size of the cell body. It has been described that the cell body can enlarge during an amoeba and macrophage challenge, indicating that size enlargement is a protective mechanism [81,82]. These cells are extremely oversized, ranging from 14- to 20-fold the size of typical yeasts grown in vitro (Figure 3 and Table 2). [83]. A mutant that overproduces titan cells is unable to be cleared from the lungs [84], suggesting that titan cells are a special morphology that enhances *C. neoformans* virulence and may be involved in latent infections that persist for several years in some humans infected with *C. neoformans* [85,86]. Titan cells are unable to be phagocytized and disseminated to the brain, but, intriguingly, they promote a 300-fold increase in the dissemination of normal size cells from lungs to the central nervous system, by a mechanism still unknown [84]. There is evidence indicating that titan cells produce daughter cells that have a normal size [59], and are resistant to oxidative and nitrosative stresses, suggesting that proliferation may be increased through a normal-size progeny that is capable of surviving inside the phagocyte and results in intracellular dissemination being easier [59,60].

## 5. Asteroid Bodies

**Figure 4:**
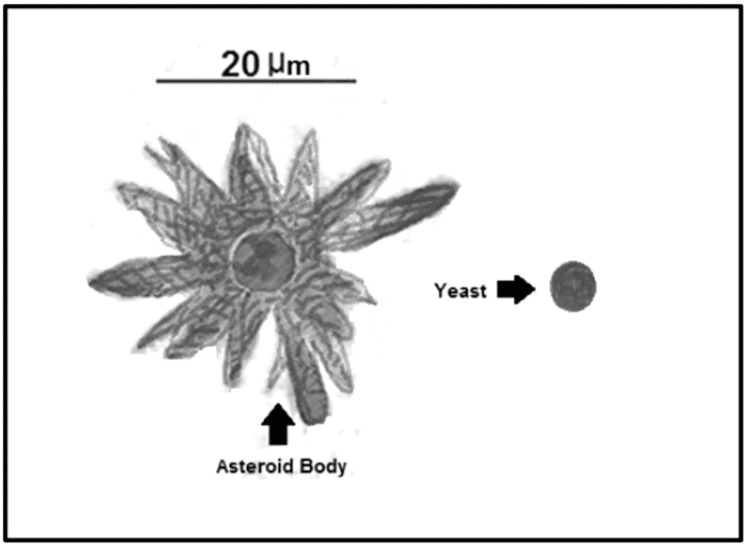
Representation of a *Sporothrix schenckii* asteroid body. Asteroid bodies can measure up to 100 µm, a bigger size when compared to the yeast form, which measures from 2 to 6 µm.

Asteroid bodies (AB) have been described in multiple fungi that are responsible for deep-seated mycosis, such as *Sporothrix, Lacazia, Candida, Histoplasma, Paracoccidioides*, and *Aspergillus* [61]. AB have been widely described in *Sporothrix schenckii,* which are found in 90% of granuloma cases associated with sporotrichosis [61]. These bodies are described as concentric layers and spikes formed around a central yeast, after a process of crystallization mediated by calcium salts and subsequent precipitation, resulting in a visible crown-like structure surrounding the yeast, which is round and different from those observed inside macrophages (smaller and cigar-shaped) [61]. Fungal molecules are not the unique components of AB; they can contain multiple host components of the immune system, such as debris from polymorphic nuclear cells and precipitated antigen–antibody complexes [87,88,89]. The AB protect the central yeast from the environment and portions of IgGs and IgMs are trapped in the external crown, suggesting an interference with the immune system. All these data support the idea that AB may act as a resistance structure, because the central yeast inside the crown is viable and capable of dividing inside the crown in vivo and in vitro conditions, forming filaments to break down the structure [61] (Figure 4 and Table 2).

## 6. Fungal Dimorphism

Dimorphism is the ability of fungal cells to switch between yeast and hypha morphologies during their life cycle. For most dimorphic fungi, both yeast and hypha forms are important for pathogenicity; such is the case with *Candida* spp. [90], because mutants that are unable to produce both morphologies are avirulent [91,92]. Morphological switch can be induced by temperature (thermal dimorphism), as in the case of the dimorphism induced once they get inside the mammalian host and reach 37 °C. Non-thermal factors can induce morphological switch as well; for example, pH for *C. albicans* or oxygen levels for the anaerobic *Mucor* spp., which can induce transition from yeast to hyphae when present [93,94]. Thermal dimorphic fungi usually grow in soil at temperatures ranging from 22 °C to 25 °C in their mycelial form and produce conidia, specialized structures that are infectious once they get inside the mammalian host cells. A morphological switch to yeast cells is critical for many fungal pathogens that grow as hyphae in the environment, such as *P. brasiliensis*, *H. capsulatum*, or *Malassezia furfur*, because many of them cannot penetrate intact skin and others can only penetrate the outer layers of the skin in their filament form (Figure 1) [95]. The interaction with the host immune system, via lectin and mannose receptors, is critical, because inside the phagocyte conidia of thermal dimorphic fungi can germinate and replicate as budding yeasts; with the help of previously formed transcripts, the adaptation to the host environment is easier, such as in *H. capsulatum* conidia or *Coccidioides* spp. spherules [96,97]. It has also been observed that during the yeast phase, genes involved in dissemination, immune evasion, intracellular survival, and virulence are upregulated, and many adhesin transcripts are produced only in the yeast phase, such as in *P. brasiliensis* gp43 [98]. In addition, there are also yeast-specialized proteins that enhance virulence, like the secreted YPS3 of *H. capsulatum*, which induces dissemination to the liver and spleen. On the other hand, the yeast-to-hyphae transition is also critical for fungal cells that grow in their yeast form in the environment, like *Candida* spp., because during infection, yeasts, pseudohyphae, and true hyphae are found [90]. *Candida* yeasts, like other pathogenic yeasts, are important for free dissemination due to their smaller size (Figure 1) [99], but hyphae invade the epithelium barrier more effectively than yeast cells, and virulence factors are expressed exclusively in the hyphal stage, such as genes that encode for proteases [90]. Although *C. albicans* yeasts are rapidly recognized and phagocytized in the bloodstream, once inside the macrophage, production of CO_2_ inside this immune cell induces the dimorphic switch from yeast to hyphae, which drills, and eventually kills macrophages, a way of subverting the host immune system [100,101]. In addition, the morphological transition from yeast to hypha in *C. albicans* has negative consequences on the ability of immune cells to produce both pro- and anti-inflammatory cytokines, as the later morphology induces low cytokine levels when compared with yeast cells [102].

## 7. Overcoming Phagocytosis

The mammalian immune system is a highly efficient machinery, set to avoid the proliferation of external agents. After fungal invasion into a mammalian host, clearance of the invading microbes relies on the host cellular immunity, mediated by elements of both the innate and adaptive immune branches. The initial response involves recognition and phagocytosis of the invading fungi by neutrophils and macrophages, as well as dendritic cells, followed by the presentation of fungal antigens to T lymphocytes. So, after triggering the adaptive immune system, in a later stage, macrophages are activated and become more specific, and conduct a second round of attack against the fungal cell. However, fungal pathogens have evolved different evasion strategies to overcome phagocytosis.

A noticeable strategy to avoid phagocytosis that some fungal pathogens have acquired is to avoid engulfing by phagocytic cells through volume expansion. This can be achieved by either cell size increase or morphogenesis. This is the case of *C. neoformans* titan cells, as already mentioned (Figure 3 and Table 2). Other fungi reach the volume increase strategy by a morphotype change, as in the dimorphic fungi *Coccidioides immitis* and *C. posadasii*. These fungi are present as hyphae in the environment, but once inside the host they form large rounded-shape spherules that reach up to 120 µm in diameter, too large to be engulfed by macrophages [103]. Similarly, the dimorphic fungi *P. brasiliensis* and *P. lutzii* can overcome phagocytosis by regulating morphogenesis. Once conidia or hyphal fragments are inhaled by the host, these fungi undergo transformation into multi-budding yeasts cells, characterized by a large mother cell (up to 30 µm in diameter) surrounded by multiple daughter cells presenting different shapes, from round to pear-like forms. The whole multi-budding morphotype can occupy an area that ranges from 75 µm^2^ to over 150 µm^2^, depending on the strain, and is difficult to engulf by macrophages [104].

In *C. albicans*, the role of dimorphism during phagocytosis is contradictory, going from a more efficient phagocytosis by macrophages for the hyphal form [105,106], to more efficient phagocytosis for the yeast cells by immortalized mouse macrophages [107]. A more recent study has shown that when macrophages are engulfing *C. albicans* hyphae, the phagocytosis rate is determined by the hypha length: hyphae with a length below 20 µm are phagocytosed at a similar rate as yeasts, while longer hyphae are engulfed at a slower rate [108]. Also, after internalization by macrophages, *C. albicans* yeast cells undergo filamentation, and germ tubes and hyphae can burst the membrane of the phagocytic cell, killing the immune cells [101].

Once activated, the phagocytic cells target the viability of the invasive fungal cell through a battery of toxic chemicals inside the phagolysosome, which includes reactive oxygen species (ROS) production, such as H_2_O_2_, which exerts cytotoxic effects on DNA, proteins, and lipids; as well as altering cellular redox homeostasis [109]. The ability of the fungal cell to overcome ROS exposure is a key determinant of its intracellular survival.

Although *C. neoformans* and *C. gatii* can avoid phagocytosis by the production of Titan cells, as previously mentioned, most of their cells remain of normal size (5 to 10 µm) and are phagocyted by macrophages. The fungus can survive inside the phagocytic cell and transmigrate across the brain–blood barrier, using the mononuclear phagocytes as a sort of “Trojan horse” to travel through the blood vessels to the brain, causing neurological disease [110]. To survive inside phagocytic cells and avoid its degradation, *C. neoformans* can reside into the phagolysosome by two main mechanisms: capsule enlargement and pH modification of the phagolysosome. The capsule is believed to allow *Cryptococcus* to resist environmental stresses such as dehydration. Once the pathogen is phagocyted, the capsule begins a process of enlargement, which lends the fungus resistance to oxidative stress without a catalase being required [111]. Purified capsular polysaccharides have been shown to present antioxidant properties, so a capsular polysaccharide-mediated scavenging of oxygen-related oxidants has been suggested as a protective mechanism against fungal killing by macrophages [111]. The increase of capsule size (and therefore the amount of polysaccharide in the capsule) would allow the fungal cell to be less vulnerable to ROS through a buffering mechanism; free radicals would preferentially affect the capsule, leaving other cell structures intact [111]. In *C. albicans*, the oxidative stress response is better understood. Two signaling pathways, Cap1 and Hog1, are responsible for the induction of key oxidative stress functions, triggering three antioxidant systems (the catalase, glutathione, and thioredoxin systems), as well as NADPH production via the pentose phosphate pathway, for the restoration of cellular redox homeostasis [112]. The first antioxidant system acting after *C. albicans* exposure to H_2_O_2_ is a detoxification mechanism led by catalase, which degrades extracellular H_2_O_2_ to water and oxygen, a reaction catalyzed by catalase (Cat1). This enzyme has a major role in H_2_O_2_ elimination in *C. albicans*, since inactivation of the *CAT1* locus significantly attenuates the decay rate of extracellular H_2_O_2_ [112]. Interestingly, it has been found that even before H_2_O_2_ exposure, *C. albicans* cells already have high levels of catalase, and therefore this detoxification mechanism seems to be of great importance in guarding against oxidative stress in this fungus [112]. The intracellular levels of H_2_O_2_ are mainly reduced by glutathione peroxidase, with the production of glutathione disulfide from glutathione. Oxidatively damaged proteins are repaired by both the glutathione system and the thioredoxin system [112]. 

High-throughput transcriptional and proteomic studies in *Paracoccidioides* spp., after macrophage phagocytosis [113,114], oxidative stress by exposure of yeast cells to H_2_O_2_ [115], and incubation of *Paracoccidioides* yeast cells with *S*-nitrosoglutathione to induce nitrosative stress [116], showed that these fungi can overcome oxidative and nitrosative stresses. In response to H_2_O_2_, *Paracoccidioides* spp. manifest activation of antioxidant enzymes: catalases, cytochrome c peroxidase, thioredoxin, and superoxide dismutases, and a metabolic shift to the pentose phosphate pathway, characterized by increased NADPH production in the cytoplasm, as an electron source for the glutathione peroxidase system, which points to restoration of the cellular redox homeostasis [115].

*H. capsulatum* is a successful intracellular pathogen, infecting both neutrophils and macrophages. After encountering the innate immune system cells, *Histoplasma* yeast cells are efficiently engulfed by macrophages, but the phagocytic cells are unable to kill the fungus [117,118,119]. Activation of macrophages elicits ROS production in response to *Histoplasma*, but pathogenic yeast cells are not killed by this reaction [120,121,122]. To date, the mechanisms eliciting *Histoplasma* intracellular survival are not fully understood. *H. capsulatum* produces extracellular catalase, and a recent study shows the importance of an external superoxide dismutase (SOD3), attached to the outside of the cell wall, in the protection of the yeast cell from superoxide [123]. A mutant lacking *SOD3* showed increased sensitivity to externally generated superoxide, by graded amounts of xanthine oxidase added to yeast suspensions in medium with increased levels of hypoxanthine. No difference in the sensitivity was observed when superoxide was internally generated by growth in the presence of paraquat. Also, the same *H. capsulatum SOD3* mutant was more susceptible to neutrophil killing than the wild-type strain [123].

To preserve redox homeostasis within the host cell, fungi can choose between enzymatic or non-enzymatic mechanisms to cope with oxidative stress. Aside the capsule, *C. neoformans* has four catalases, two superoxide dismutases, glutathione peroxidases, thioredoxin proteins, protein kinase C and the inositol phosphosphingolipid-phospholipase C1 to manage the oxidative environment inside the macrophages [124,125,126,127,128].

Upon ingestion by alveolar macrophages, *A. fumigatus* conidia are processed within the phagolysosome, most likely with the contribution of ROS, as murine alveolar macrophages unable to produce NADPH oxidase-mediated ROS do not kill *A. fumigatus* conidia [129]. To overcome this, it is thought that fungal superoxide dismutases reduce ROS stress by detoxifying superoxide anions [130].

Similarly to *Aspergillus*, *Candida* spp. possess catalases, superoxide dismutases, and glutathione peroxidases that protect the fungus against respiratory burst [131,132,133]. This powerful antioxidant defense system has also been described for a wide range of fungi, including *H. capsulatum* and *P. brasiliensis*. Furthermore, in the latter, it has been shown that catalase activity is induced when yeast cells are exposed to H_2_O_2_ [134,135].

*Cryptococcus* can start a process of intracellular microenvironment modification by permeating the membrane of the phagolysosome and increasing the pH from 4.3 to around 5.4, which is closer to its optimal pH for growth [136]. In fact, the degree of damage to the phagolysosome membrane presents a direct correlation to fungal growth inside the macrophage. The mechanism by which *C. neoformans* promotes phagolysosome membrane damage remains unknown, although some hypotheses have been discussed and are reviewed by [137]. The permeation of the phagolysosome membrane by *Cryptococcus* has been found to be related to naive macrophages. When macrophages are activated by IFN-γ, they show considerable improvement in fungal killing, so this fungal survival mechanism might be more important during the initial infective process for *Cryptococcus* [138]. Similarly, *H. capsulatum* resides in neutralized mature phagosomes, and the mechanism for phagosome neutralization points to permeabilization of the phagosomal membrane via a saponin-like protein (Cbp1), which is secreted into the phagosomal space [139,140]. In *C. albicans*, the microenvironment manipulation could depend on the engulfed morphotype by the macrophage. A recent study suggests that *C. albicans* yeast cells neutralize the phagolysosome by metabolic changes, rather than membrane permeabilization [141]. Once phagocyted, *C. albicans* yeasts can differentiate into filamentous hyphae, which elongate and pierce the macrophage membrane, allowing fungal escape [101]. This morphological switch requires a neutral pH, and *C. albicans* yeasts would manipulate the phagosome microenvironment by extrusion of ammonia, as a byproduct of a switch in the amino acid catabolism. This would be reached via STP2, a transcriptional regulator of amino acid permease genes, and therefore of amino acid transport [141]. Yeast cells of a *C. albicans* mutant defective in *STP2* are unable to form hyphae within the macrophage and fail to escape the phagocytic cell, but neutralization of the phagosome restarts their ability to filament and to escape from macrophages [141]. Another study proposes that *C. albicans* hyphae, internalized by macrophages, avoid acidification (and other antimicrobial arsenals contained in the lysosome), through manipulation of the phagosome maturation, by altering the dynamic of Rab14, a GTPase of the host cell, whose transient activity is involved in phagosome maturation, promoting the acquisition of phagosome maturation markers LAMP1 and lysosomal cathepsin to form a fully bioactive lysosome [142]. When *C. albicans* yeast-locked cells, wild-type yeast cells, and hyphae of different lengths were phagocyted, authors observed retention of Rab14 in the macrophage membrane, which impaired the fusion of lysosomes to the phagosome. The retention of Rab14 was directly proportional to the length of the engulfed hypha, therefore impairing the phagosome maturation [142].

## 8. Deceiving the Humoral Immune Response

Regarding the humoral response against fungi, the antimicrobial peptides and the complement system are two major, evolutionary conserved, mechanisms found as the first line of defense against microbes. Antimicrobial peptides are small peptides of 20 to 50 amino acid residues, varying in length, sequence, and structure. They are genetically coded by only one gene and, despite posttranscriptional modifications, are usually amphipathic and cationic molecules [143,144]. These soluble molecules kill or limit the growth of organisms by membrane permeabilization of microbial cells, and by inactivation of cytoplasmic targets therein [145]. Alongside antimicrobial activity, antimicrobial peptides also act as immunomodulators by upregulating TNFα, thus promoting migration of neutrophils and monocytes to the site of infection, and by chemoattraction of immature dendritic and T cells to modify the adaptive immune response [146]. 

One of the *Candida* first-line strategies to escape from these immune molecules is the secretion of proteins that inactivate the antimicrobial peptides. Secreted aspartyl proteases (Saps) Sap9 and Sap10 inactivate histatin 5 by cleavage, resulting in a complete loss of the killing properties of this salivary peptide [147]. *C. albicans* also secretes the Msb2 glycodomain upon cleavage of the Msb2 plasma membrane protein. This glycodomain inactivates a wide range of antimicrobial peptides, including LL-37, histatin 5, hNP-1, and hBD1, by binding tightly to them [148,149]. Histatin 5 accumulates inside the fungal cell, inducing oxidative stress , and ATP efflux. In this regard, it has been shown that the polyamine efflux transporter Flu1 mediates efflux of histatin 5 in *C. albicans*, reducing its toxicity [150]. Mediation of resistance to basal concentrations of antimicrobial peptides has been linked to high-osmolarity glycerol (HOG) pathway in *C. albicans*. Physiological levels of histatin 5 activated the mitogen-activated protein kinase (MAPK) Hog1, and β-defensins were shown to trigger Hog1 activity by interaction with the upstream MAPK Pbs2, to orchestrate a cell damage compensatory response by downregulation of ROS production and of ATP efflux [151,152,153]. Ssd1, a RNA-binding protein of the regulation of Ace2 and morphogenesis (RAM) pathway, along with its downstream transcription factor Bcr1, enhance antimicrobial peptide resistance by maintaining mitochondrial integrity, and by reducing membrane permeabilization, ever since the RAM pathway is involved in cell wall integrity, Ssd1 could control cell wall and membrane adaptation to reduce β-defensin activity [154,155].

On the other hand, complement activation, by either classical, alternative, or lectin pathways, leads to opsonization and assembly of the membrane attack complex. In fungal species, the presence of the fungal cell wall makes it challenging for this complex to effectively disturb fungal viability. However, proper opsonization enhances the phagocytic process and further killing of the organism by neutrophils and macrophages [156]. Peltz et al. [157] developed a computational genetic mapping program with advanced features that allowed for the identification of genetic factors affecting survival in a murine genetic model of hematogenous *C. albicans* infection; and their results suggested that the genetic variation in components of the complement pathway, specifically C1q, C1r, and C1s, could compromise the survival of the infected mice. Furthermore, these results were confirmed in vivo and gave rise to a genetic model wherein the interaction of C5 and C1r/s alleles accurately predicted survival after *Candida* infection [157]. The significance of C3 and C5 for the anti-*Candida* humoral response has been previously demonstrated, since mice deficient in either of these components exhibited more susceptibility to disseminated candidiasis [158,159]. Thus, to survive, fungal pathogens have developed strategies to cope with this immune threat. Once the complement system has been activated, the progression of the signaling cascade is controlled by inhibitors widely distributed in body fluids or plasma, and also attached to the surface of host cells to avoid damage [160]. Fungi take advantage of the fluid phase inhibitors, binding them on the fungal surface to disguise the host and thereby eluding complement activation. For instance, pH-regulated antigen 1 (Pra1) is a *C. albicans*-secreted surface protein that binds human plasma proteins Factor H and Factor H-like 1 (FHL-1) [161]. Since these proteins control the activation of the complement alternative pathway by acting as cofactors for factor I, a serine protease in charge of cleaving C3b into inactive C3b (iC3b) [162]. Moreover, Pra1 binds to C4b-binding protein (C4BP), which acts as a cofactor for factor I-mediated cleavage of C4b, preventing the formation of the classical and lectin pathways C3 convertase (C4bC2b) at the fungal surface, and consequently blocking further progression of the complement cascade, reducing the generation of proinflammatory products, and limiting C4b and C3b surface opsonization, thus leading to diminished uptake by host macrophages [163]. Similar effects in blocking complement activation are exerted by Pra1 when secreted by both yeast and hyphae [26,161]. Additionally, attachment of some surface or secreted fungal proteins, like Pra1, to plasminogen may result in the conversion of this plasma protein into its proteolytically active form, plasmin, which cleaves fibrinogen and thus aids in destroying the host extracellular matrix [27]. Similarly to Pra1, phosphoglycerate mutase 1 (Gmp1) and glycerol-3-phosphate dehydrogenase 2 (Gpd2) are fungal surface proteins that bind to factor H, FHL-1, and plasminogen, exerting effects comparable to this reported for Pra1; however, Gmp1 does not bind C4BP and Gpd2 is not secreted, which may have a role in fungal adhesion and direct contact between the fungus and human endothelial and epithelial cells [160,164]. Another factor H- and C4BP-binding fungal protein that has been reported in *C. albicans* is the high-affinity glucose transporter 1 (Hgt1), proven to act as a complement inhibitor [165]. Despite the fact that similar complement-evasive behavior has been reported in *Aspergillus*, the identity of the specific proteins resembling those found in *Candida* has not yet been established. Nevertheless, binding of this mold to factor H, FHL-1, plasminogen, factor H-related protein 1, and C4BP has already been demonstrated and is morphology-dependent [166,167]. In addition to this fungal strategy to counteract the immune response, it has been reported in *C. albicans* that Sap1, Sap2, and Sap3 degrade host complement components C3b, C4b, and C5, and also inhibit terminal complement complex formation [30].

*A. fumigatus* hyphal morphology binds lower amounts of the complement regulators, but secretes proteases, such as Alp1, that degrade complement proteins C3, C4b, C5, and C1q, inhibiting complement activation [31]. Henwick et al. demonstrated by quantitative flow cytometry that the highly pathogenic species *A. fumigatus* and *A. flavus,* unlike less pathogenic species such *A. glaucus*, *A. nidulans*, *A. niger*, *A. ochraceus*, *A. terreus*, *A. versicolor*, and *A. wentii*, bound fewer C3 molecules per unit of conidial surface area, suggesting that putative complement recognition sites on the conidial surface of *A. fumigatus* and *A. flavus* are masked to minimize the stimulus for complement activation [168]. Although the detailed mechanism of how melanin decreases complement deposition remains unclear, it has been confirmed that a lack of enzymes involved in melanin synthesis leads to increased C3 binding on the conidial surface [169,170,171]. Fungi producing melanin-like pigments, such as *P. brasiliensis,* also exhibit the same phenomenon. Yeast melanization causes significant interference with the binding of cell wall components to lectin receptors on macrophages, decreasing the efficiency of complement-dependent phagocytosis [172].

*Aspergillus* spp. not only acquires complement inhibitors from the host, but also produces, and releases, its own soluble factor to inhibit complement activation and opsonization. This complement inhibitor (CI) selectively abolishes activation of the alternative pathway, and plays a role in C3-dependent phagocytosis [173].

In *Blastomyces dermatitidis* the surface protein BAD1, an adhesin that binds the yeast to CD14 and complement receptors, was suggested to play a role in complement evasion by engaging C3 sites on cell wall glucans and thus preventing C3 deposition [174].

It has been established for *C. albicans* that cell surface heat shock proteins Ssa1 and Ssa2 are required for histatin 5 binding to the fungal cell wall, and this is necessary for subsequent intracellular translocation [175]. These proteins are also needed for human β-defensin 2 (HBD2) candidacial activity [176]. Given the similarities existing between HBD2 and histatin 5 in terms of ion selectivity and energy requirements, it is possible that the translocation mechanism for HBD2 could be the same as for histatin 5. However, the resistance of *C. glabrata* to HBD2 seems not to be the result of lacking Ssa proteins [177].

Melanin also plays an important role in defending the fungus against antimicrobial peptides. Binding of these peptides to melanin abrogates their activity, as it was demonstrated that melanized cells of *C. neofromans* were less susceptible to killing by three antimicrobial peptides: a defensin, a protegrin, and a magainin [178].

## 9. Are You Trapped? Be Like *Cryptococcus*!

Neutrophils constitute a primary defense against fungal infections, rapidly recruited to the site of infection in response to chemotactic factors released by fungal or host cells. As innate immune cells, neutrophils use different strategies to limit the infection, such as the well-known degranulation process and phagocytosis, but also the more recently described formation of extracellular traps (NETs) [179]. In a process called netosis, a kind of cell death, neutrophils shape a trap by extruding its nuclear DNA fibers to the extracellular space, along with antimicrobial molecules derived from its granules and cytosol. NETs retain and kill microorganisms while hampering tissue damage caused by neutrophil microbicidal molecules associated with these traps. Netosis occurs upon contact with a variety of pathogens or with many host factors, such as activated platelets, inflammatory stimuli, or chemical compounds [180]. Among fungi, *C. albicans, A. fumigatus, A. nidulans*, and *C. gattii* induce NETs formation and are susceptible to its fungicidal or fungistatic effects [34,181,182,183]. Nevertheless, Rocha et al. demonstrated that *C. neoformans* capsular GXM avoids the formation and fungicidal effect of NETs, thus constituting an escape mechanism evolved by this pathogen (Table 2) [58]. There is also evidence that hydrophobin RodA helps *A. fumigatus* conidia to evade NET induction (Table 1) [34,35].

## 10. Overcoming Ion Starvation or Nutritional Immunity

Due to its redox capacity, iron is a cofactor of cytochromes, oxygen binding molecules, and a variety of enzymes; it can also be a threat to tissues, catalyzing the conversion of hydrogen peroxide to free-radical ions that attack cellular membranes, DNA, and proteins. This is the reason why, despite the high amount of iron in the bloodstream, this metal is mainly sequestered by hemoglobin or bound to plasma transferrin [184]. Such seizure of iron provides the host with protection against iron-catalyzed free radical generation and provides a sort of nutritional immunity against iron-dependent pathogens, such as *C. albicans* [185]. Additionally, it has been established that one defense mechanism against invading pathogens involves acute declines in the levels of iron in serum, a decay in serum turnover, retention of iron within reticuloendothelial cells, and decreased intestinal absorption [186]. *Candida* strategies for iron acquisition can be grouped into three types: (i) hemolytic activity; (ii) binding to iron-binding host molecules and; (iii) usage of siderophores. The first targets the release of hemoglobin from red blood cells to further bind the hemoglobin to the fungal cell surface, via a Rbt5 receptor, and then endocytoses this complex to finally release the Fe^2+^ by the heme oxidase Hmx1 [187,188,189]. For the second, it has been suggested, by in vitro experiments, that *C. albicans* expresses both transferrin and ferritin receptors that allow it to utilize host iron-binding proteins as a source of iron (Table 1) [28,29]. Fe^3+^ derived from transferrin is reduced to Fe^2+^ by a cell surface-associated reductase, and then oxidized again at the time it is imported to the cytoplasmic space by a multicopper ferroxidase/iron permease complex, with the ferric permease Ftr1 being the one expressed under low iron conditions [190,191,192]. Ferritin binding is mediated by the hypha-specific cell surface Als3 protein and further processed via the same iron uptake system described for transferrin [29]. Finally, the third strategy focuses on aiming siderophores, high-affinity Fe^3+^ chelators most likely from exogenous origin imported by *Candida* via the Sit1 siderophore importer [193].

To access iron, *A. fumigatus* employs endogenous siderophores for iron scavenging and, although only this pathway is required for virulence, it also possesses a reductive iron acquisition (RIA) system comprising a permease and a multicopper oxidase for high-affinity ferric import (FtrA/FetC) [194]. *H. capsulatum* produces siderophores, secretes gamma-glutamyltransferase and glutathione-dependent ferric reductase, and also relies on RIA (Ftr1/Fet3) [195,196]. *C. neoformans* uses the RIA (Ctf1/Cfo1), secreted melanin, and 3-hydroxyanthranilic acid as iron reductants and Cig1 protein for hemoglobin iron extraction [197,198].

Besides iron sequestration, the most well-studied example of nutritional immunity, the struggle for non-iron transition metals during infection is a subject that has recently received significant attention. Zinc is the second most abundant transition metal in humans, functioning as a protein cofactor and displaying both catalytic and structural roles. It is critically important for proper immune function, but there is evidence suggesting that zinc sequestration by the host during infection is a nutritional immunity strategy to impede microbial growth [32,33]. Citiulo et al. determined that, similarly to the siderophore-mediated iron acquisition, the *C. albicans* hypha utilizes secreted Pra1 as an extracellular zinc scavenger, a so-called zincophore, and the Zn–Pra1 complex is then bound to the fungal surface by zinc transporter Zrt1. Therefore, Pra1 mediates endothelial damage by scavenging host zinc (Table 1) [199].

Copper is a metal ion that functions as a structural and catalytic cofactor for enzymes involved in energy generation, oxygen transport, cellular metabolism, and iron acquisition [200]. Cell surface metalloreductases reduce Cu^2+^ to Cu^1+^ in *Saccharomyces cerevisiae* and then copper is internalized by the high-affinity transporters Crt1 and Crt3 [201,202,203]. Similar genes for those encoding proteins involved in metal homeostasis in *S. cerevisiae* and *C. albicans* were found in the genomes of *P. brasiliensis*, *C. neoformans* var. *grubii*, and *C. gattii*, suggesting that the mechanisms involved in overcoming ion starvation are conserved in those fungi [196]. 

## 11. The Role of Fungal Biofilm Formation during Interaction with the Host Immune Response

Biofilms are functionally heterogeneous, three-dimensional structures formed by microbes encapsulated in a matrix of self-produced extracellular polymeric molecules that could adhere to biotic or abiotic surfaces. In biofilms, single cells might differentiate to gain specialized properties or functions [62,63]. It has been estimated that up to 80% of all microorganisms grow as biofilms rather than as planktonic cells, and this mode of growth allows them to survive in hostile environments [204,205,206]. Regarding fungal biofilm interaction with the immune system, there is supporting evidence that the presence of adherent peripheral blood mononuclear cells (PBMCs) enhances the ability of *C. albicans* to form biofilms, most likely mediated by a soluble factor secreted by PBMCs, and without apparent phagocytic process [207]. Likewise, it was found that the presence of the proinflammatory cytokine IL-17A boosted *C. albicans* biofilm formation in vitro [64]. Mature biofilms were shown to be resistant to the attack of neutrophils and to avoid ROS triggering without compromising the leukocytic viability. These biofilm-friendly features were mediated by the β-glucans found in the biofilm extracellular matrix, representing an important innate immune evasion tool [208].

Likewise, *Aspergillus* biofilms are more resistant to the antifungal activity of neutrophils and PBMCs than planktonic cells. Notably, they can be mediated by gliotoxin, a fungal virulence factor that promotes cellular apoptosis [65,66]. When forming biofilms, *C. neoformans* also shows resistance to oxidative stress but remains vulnerable to β-defensins. Cationic β-defensin 1 and β-defensin 3 interact with the negatively charged biofilm surface to exert its antimicrobial function. Nonetheless, melanized biofilms were significantly less susceptible to antimicrobial peptides than non-melanized biofilms [209]. These data support the observation that biofilm formation might be an adaptation to survive the clearance mechanisms orchestrated by the host.

## 12. Other Fungal Strategies to Avoid Immune Sensing

In *Pneumocystis carinii* it has been demonstrated that a glycosylated surface protein, known as gpA, is involved in binding to host proteins, fibronectin, and vitronectin, enhancing fungal attachment to alveolar epithelial cells, and promoting colonization, and is recognized by innate immune cells in a mannose-dependent way [210,211]. In this context, it has been demonstrated that this fungus secretes gpA to favor competitive binding of the secreted protein instead of the surface protein to the mannose receptor, leading to a blockage of phagocytosis by alveolar macrophages [212]. Interestingly, some studies in mice genetically impaired in the macrophage mannose receptor have shown that these mice are able to clear *Pneumocystis* from the lungs, thus suggesting compensatory mechanisms via other immune receptors [213].

For *C. posadasii*, a fungal respiratory pathogen, a metalloproteinase (Mep1) has been found to be secreted during endospore differentiation. Mep1 digests the spherule outer wall glycoprotein, an adhesin that contributes to virulence, resulting in the prevention of host recognition and further phagocytosis of fungal endospores, contributing to the ability of the pathogen to persist within the host [98,214].

It has been shown that *C. albicans* and non*-albicans* species, such as *C. tropicalis*, *C. krusei*, *C. parapsilosis*, and *C. guilliermondii*, are efficiently phagocytosed; nonetheless, they are also able to filament inside and outside the macrophage to avoid phagocytosis and limit the recruitment of additional macrophages or to destroy the phagocytic cell [215,216]. *Candida* species unable to undergo dimorphic transition have also developed strategies to escape phagocytosis. *C. lusitaniae* escapes from macrophage activities by producing cell chains and *C. glabrata* proliferates inside macrophages, resulting in host cell lysis [217,218]. *C. glabrata* can block phagolysosome formation and acidification because this pathogen does not acquire cathepsin D, a lysosomal acidic enzyme allowing fungal replication [219]. Furthermore, the phosphoinositide 3-kinase protein produced by *C. glabrata* seems to be related to the modulation of the phagosome maturation, since mutants lacking this protein displayed a larger number of acidic phagosomes [220].

## 13. Concluding Remarks

It is clear that the immune system plays a major role in defense mechanisms against fungal pathogens, and this groups of organisms have evolved strategies to curb or exploit the immune response to their benefit. The classical approach to treat fungal infections is antifungal drugs, which target the plasma membrane, synthesis of ergosterol, or β-1,3-glucan. Although the current therapeutic choices are still adequate for the control of most of the patients affected with a disease caused by fungi, the increment in the number of fungal isolates resistant to one or multiple drugs is a major concern. This has driven the scientific community to look for new therapeutic alternatives to expand the repertoire of antifungal drugs. The search for these new molecules should take into account not only the ability to target cell viability but also the expression of virulence factors and the mechanisms to evade the immune response. Molecules capable of blocking any of the strategies discussed here would increase, with no doubt, infected patients’ chances of a full recovery. At first glance, this could be of little help to immunocompromised patients, but the combination of these molecules with immunostimulants might be useful in the management of opportunistic mycoses.

## Figures and Tables

**Table 1:** Molecules involved in host immune evasion. The table summarizes the different molecules associated to host immune evasion. Their function and effect on the immune system are briefly described.

Molecules Involved in Host Immune Evasion	Function	Effect on the Human Immune System	Fungi Where They Have Been Described
**Mannans**	Proper cell wall architecture	Shielding of β-1,3-glucans from Dectin-1 receptor [13,14,15,16,17,18,19,20]	*Candida spp, H. capsulatum yeast* [13,14,15,16,17,18,19,20]
**Melanin**	Mechanical strength of the cell wall, enzymatic degradation resistance and UV protection [24]	Resistance to phagocytosis [9,24,25]	*C. neoformans*, *Paracoccidioides brasiliensis*, and *Sporothrix schenckii* [24]
**α-1,3-Glucan**	Proper cell wall structure and architecture [21]	Shielding of β-1,3-glucans from Dectin-1 receptor [21]	*H. capsulatum* yeast [21]
**Endo β-1,3-glucanase**	Trimming of β-1,3-glucan segments exposed on the fungal cell surface [23]	Reduced recognition of the yeast via Dectin-1 and as a consequence, a reduction in stimulation of proinflammatory cytokines [23]	*H. capsulatum* yeast [23]
**Transferrin Receptors**	Utilizing host iron-binding proteins as a source of iron [26,27]	Overcoming the host nutritional immunity of iron by the host [28,29]	*C. albicans* [28,29]
**Pra 1**	Scavenger of host zinc [30,31]	Overcoming the nutritional immunity of zinc by the host [32,33]	*C. albicans* [32,33]
**Rod A**	Cell wall hydrophobin	Diminished host NETs formation [34,35]	*A. fumigatus* [34,35]

**Table 2:** Fungal structures involved in the host immune evasion.

Structures and Architectural Changes Associated in Host Immune Evasion	Function	Effect on the Human Immune System	Fungi Where They Have Been Described
**Fungal Capsule**	Protection form environment and source of virulence factors [51]	Anti-phagocytic properties [56], suppression of T lymphocyte proliferation [56,57], induction of the anti-inflammatory cytokine IL-10, reduction in pro-inflammatory cytokines [56] and survival inside macrophage environment [55]. NETs formation is impeded by the capsular component GXM [58]	*C. neoformans* [53,58]
**Titan-cell Formation**	Protection from hostile environments [59,60]	Evasion of phagocytosis and resistance to oxidative and nitrosative stresses [59,60]	*C. neoformans* [59,60]
**Asteroid Bodies**	Resistance structure that protects the central yeast from the environment	Trapping of IgGs and IgMs, interfering with the proper immune system action [61]	*Sporothrix* spp.; *Lacazia* spp.; *Candida* spp.; *Histoplasma* spp.; *Paracoccidioides* spp.; *Aspergillus* spp. [61]
**Biofilm Formation**	Adhesion on biotic or abiotic surfaces, survival on hostile environments [62,63]	Resistance to neutrophil attack, avoids ROS triggering, and increases resistance to the antifungal activity of PBMNCs	*Candida* spp.; *Aspergillus* spp.; *C. neoformans* [64,65,66]
